# G-Protein-Coupled Estrogen Receptor Agonist Suppresses Airway Inflammation in a Mouse Model of Asthma through IL-10

**DOI:** 10.1371/journal.pone.0123210

**Published:** 2015-03-31

**Authors:** Masamichi Itoga, Yasunori Konno, Yuki Moritoki, Yukiko Saito, Wataru Ito, Mami Tamaki, Yoshiki Kobayashi, Hiroyuki Kayaba, Yuta Kikuchi, Junichi Chihara, Masahide Takeda, Shigeharu Ueki, Makoto Hirokawa

**Affiliations:** 1 Department of General Internal Medicine and Clinical Laboratory Medicine, Akita University Graduate School of Medicine, 1-1-1 Hondo, Akita, 010–8543, Japan; 2 Department of Clinical Laboratory Medicine, Hirosaki University Graduate School of Medicine, 5 Zaifu-cho, Hirosaki 036–8562, Japan; 3 Division of Dentistry and Oral Surgery, Akita University Graduate School of Medicine, 1-1-1 Hondo, Akita, 010–8543, Japan; 4 Nagareyama Tobu Clinic, 909–1 Nazukari, Nagareyama City, Chiba, 270–0145, Japan; 5 Department of Otolaryngology, Kansai Medical University, 2-5-1 Shin-machi, Hirakata City, Osaka, 573–1010, Japan; 6 Soseikai General Hospital, 101 Shimotoba Hiroosacho, Fushimi-ku, Kyoto City, Kyoto, 612–8473, Japan; 7 Department of Cardiovascular and Respiratory Medicine, Akita University Graduate School of Medicine, 1-1-1 Hondo, Akita, 010–8543, Japan; Baylor Institute for Immunology Research, UNITED STATES

## Abstract

Estrogen influences the disease severity and sexual dimorphism in asthma, which is caused by complex mechanisms. Besides classical nuclear estrogen receptors (ERαβ), G-protein-coupled estrogen receptor (GPER) was recently established as an estrogen receptor on the cell membrane. Although GPER is associated with immunoregulatory functions of estrogen, the pathophysiological role of GPER in allergic inflammatory lung disease has not been examined. We investigated the effect of GPER-specific agonist G-1 in asthmatic mice. GPER expression in asthmatic lung was confirmed by immunofluorescent staining. OVA-sensitized BALB/c and C57BL/6 mice were treated with G-1 by daily subcutaneous injections during an airway challenge phase, followed by histological and biochemical examination. Strikingly, administration of G-1 attenuated airway hyperresponsiveness, accumulation of inflammatory cells, and levels of Th2 cytokines (IL-5 and IL-13) in BAL fluid. G-1 treatment also decreased serum levels of anti-OVA IgE antibodies. The frequency of splenic Foxp3^+^CD4^+^ regulatory T cells and IL-10-producing GPER^+^CD4^+^ T cells was significantly increased in G-1-treated mice. Additionally, splenocytes isolated from G-1-treated mice showed greater IL-10 production. G-1-induced amelioration of airway inflammation and IgE production were abolished in IL-10-deficient mice. Taken together, these results indicate that extended GPER activation negatively regulates the acute asthmatic condition by altering the IL-10-producing lymphocyte population. The current results have potential importance for understanding the mechanistic aspects of function of estrogen in allergic inflammatory response.

## Introduction

Asthma is known to be a sexually dimorphic disease in terms of severity; women have more severe asthma than men with increased airway hyperresponsiveness (AHR) [1,2,3]. Indeed, experimental evidence including ours indicates that female mice are more susceptible to development of allergic airway inflammation, AHR, and airway remodeling [4,5]. A recent clinical study using cluster analysis revealed a female-dominant phenotype, indicating the heterogeneity of asthma and different pathophysiology in female asthmatics [6].

A role for estrogen in modulating asthma is deduced from the natural history of asthma. Coincident with the onset of puberty and increasing levels of circulating estrogen, asthma becomes significantly more common in women than in men, particularly during the reproductive years and pregnancy [7]. Another observation regarding the contribution of estrogen is that female asthmatics can be affected by pregnancy, menstruation cycle, menopause, and hormone replacement therapy [8]. Many epidemiological and clinical studies have shown that estrogen likely contributes to disease severity and development of asthma, although the results are not consistent. In contrast, several studies have indicated that supplemental estrogen is successfully used as a steroid-sparing agent in women with severe asthma [9,10]. The influence of estrogen has been investigated in animal models of asthma with both favorable and unfavorable results [11]. Therefore, understanding the functional mechanism of estrogen in asthmatics is potentially important to achieve future personalized treatment.

Estrogen has a multitude of biological effects not only on the female reproductive system but also on the immune system. The actions of estrogen have been traditionally described as occurring through one of the two classical nuclear estrogen receptors, estrogen receptor (ER) α and ERβ, which function as ligand-dependent transcription factors that bind directly to estrogen response elements in the promoter regions of genes. In addition to the long-term regulation of gene expression, estrogen has also been shown to meditate many rapid biological responses. An estrogen-binding site was found on the cell membrane [12,13], and G-protein-coupled receptor (GPCR) was identified as an estrogen-binding membrane receptor. G-protein-coupled estrogen receptor (GPER) is abundantly expressed not only in the brain and cardiovascular systems but also in the lungs [14,15]. In addition to the fact that ERs and GPER possess different signaling mechanisms, their actions are thought to be independent by several measures of difference, such as expression, binding affinity to estrogen, and biological functions. A GPER-selective agonist has been linked to a variety of pathological and physiological events regulated by estrogen action, including female reproductive cancer and the renal and cardiovascular systems [16]. To date, several studies have indicated the immunoregulatory functions of GPER [17,18,19,20,21,22], although the roles of GPER in allergic inflammatory diseases have yet to be elucidated.

Given this background, we aimed to investigate the role of GPCR in asthmatic mice using GPER-specific agonist G-1. Our data indicated that extended GPER activation negatively regulated the Th2-mediated airway inflammatory response in an interleukin (IL)-10-dependent manner.

## Materials and Methods

### Animals

Female BALB/c, C57BL/6, and IL-10 KO mice at 8–10 weeks of age were purchased from Charles River Japan, Inc. (Yokohama, Japan). These mice were maintained on ovalbumin (OVA)-free diets. All experimental animals used in this study were housed under constant temperature and light cycles, and under a protocol approved by the Institutional Animal Care and Use Committee of Akita University Graduate School of Medicine and Faculty of Medicine.

### Sensitization and airway challenge

**Fig 1:**
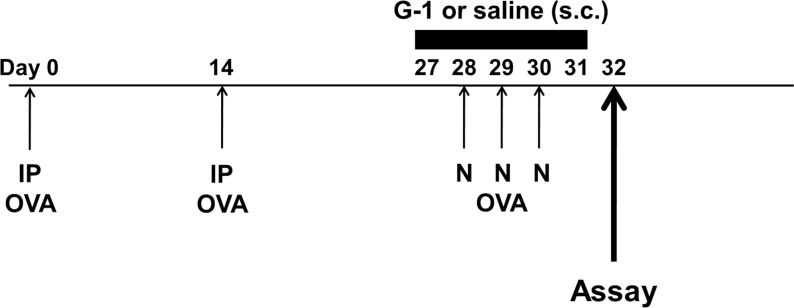
Experimental protocols and immunofluorescence staining demonstrated GPER expression. Mice were sensitized by two intraperitoneal injections (IP) of OVA/alum and then subjected to three consecutive days of aerosolized OVA challenge (N). To evaluate the effect of G-1 on airway hyperresponsiveness (AHR) and airway inflammation, mice received subcutaneous injections (s.c.) of G-1 (5 μg daily) from Days 27 to 31.

Mice were immunized by intraperitoneal injection of 20 μg OVA (Grade V; Sigma-Aldrich, St. Louis, MO) emulsified in 2.25 mg of alum (Imject Alum; Pierce, Rockford, IL) in a total volume of 100 μl on Days 0 and 14. Mice were challenged via the airways with 1% OVA in saline for 20 min on Days 28, 29, and 30 by ultrasonic nebulization (Fig 1). Lung resistance and dynamic lung compliance to methacholine (Sigma-Aldrich) were assessed 48 hours after the last challenge, and tissues and cells were obtained for further assays [23,24].

### Administration of G-1

G-1 (Cayman Chemical Company, Ann Arbor, MI) was purchased [25]. Mice received subcutaneous injections of G-1 5 μg daily [26,27], from Days 27 to 31 (Fig 1). G-1 was dissolved in dimethyl sulfoxide (DMSO) (Sigma-Aldrich) and adjusted to a density of 5 μg/100 μL/mouse.

### Evaluation of AHR

Lung resistance and dynamic lung compliance to methacholine were measured using an invasive system, as previously described [28]. Briefly, mice were deeply anesthetized, and tracheotomies were performed. The mice were then placed in an Elan Series Mouse RC Site chamber (Buxco Electronics, Wilmington, NC) and ventilated mechanically. Once baseline data were established, saline and an increasing dose of nebulized methacholine were administered. At each dose, airflow changes in the sealed chamber and pressure changes in the airway were analyzed with BioSystem XA software (Buxco Electronics) for 3 minutes. Lung resistance and dynamic lung compliance for each methacholine dose were expressed as a percentage change from baseline level.

### Collection of bronchoalveolar lavage fluid and measurement of cytokines

The lungs were lavaged through the tracheal tube with saline (1 mL, 2 times, 37°C). The volume of collected bronchoalveolar lavage (BAL) fluid was measured in each sample, and the number of BAL cells was counted. Cytospin slides were stained with May-Giemsa, and at least 300 cells were differentiated in a blinded fashion under light microscopy. Cytokine concentrations in the BAL fluid supernatants were measured by means of enzyme-linked immunosorbent assay (ELISA). Colorimetric measurement was performed according to the manufacturer’s instructions. The minimum detectable doses (MDD) are 2 pg/mL of mouse IL-4 and interferon gamma (IFN-γ), 7 pg/mL of IL-5, 1.5 pg/mL of IL-13, and 3 pg/mL of eotaxin, respectively (R&D Systems, Minneapolis, MN).

### Measurement of serum levels of total and OVA-specific immunoglobulin E (IgE) antibodies

Total and OVA-specific IgE antibody levels were measured by means of ELISA (Bethyl Laboratories, Inc., Montgomery, TX) 48 hours after the last airway challenge [29].

### Histological evaluation

The lungs were inflated through a tracheal tube with 2 mL of air and fixed in 10% formalin, and lung tissue was embedded in paraffin. Tissue sections with a thickness of 4 μm were affixed to microscope slides and deparaffinized. The slides were stained with hematoxylin and eosin (H&E) and examined under light microscopy to detect inflammatory cell infiltrates. In H&E lung sections, the numbers of inflammatory cells per square millimeter in the peribronchial areas were analyzed using the National Institutes of Health (NIH) Image Analysis system [30]. Serial sections were also stained with Periodic acid-Schiff (PAS) and Masson trichrome (MT). For histopathologic analysis, sample regions were randomly selected in a coded manner.

### Splenocyte culture for cytokine production

The culture of splenocytes was performed as described previously [31]. Briefly, on Day 32, mice were sacrificed, the spleens were excised, and the splenocytes were disaggregated. Splenic mononuclear cells were isolated by density gradient using Histopaque-1083 (Sigma-Aldrich), and the washed cells were resuspended at 8 × 10^6^/ml in complete medium consisting of Roswell Park Memorial Institute 1640 (RPMI 1640) (Life Technologies, Carlsbad, CA) with 10% heat-inactivated fetal bovine serum (FCS) (Life Technologies), 50 ng/mL phorbol myristate acetate (PMA) (Sigma-Aldrich), and 10 mM A23187 (Sigma-Aldrich). The cells were then cultured for 2 days at 37°C in a 5% CO_2_ humidified atmosphere. For the cytokine assay, the culture supernatants of the cells were collected at 2 days. The levels of IL-10 in the supernatants were measured using ELISA.

### Flow cytometry

Splenocytes were prepared from the C57BL/6 mice after sensitization and airway challenge with OVA in the presence or absence of G-1 administration. Briefly, on Day 32, splenic mononuclear cells were isolated as shown above. The cells were pre-incubated with an anti-mouse FcR blocking reagent and then incubated at 4°C for cell surface staining with a combination of fluorochrome-conjugated antibodies: *1)*, PE-Cy5-conjugated anti-TCRβ (BioLegend, San Diego, CA), PE-Cy7-conjugated ant-NK1.1 (BioLegend), PE-CF594-conjugated anti-CD8a (BD Biosciences, San Jose, CA), and Alexa Fluor 488-conjugated anti-CD4 (BioLegend). Then, the cells were washed, permeabilized with BD Cytofix/Cytoperm (BD Biosciences), and stained with PE-conjugated anti-IL-10 (BioLegend) to detect intracellular IL-10. *2)*, PE-Cy5-conjugated anti-TCRβ (BioLegend, San Diego, CA), PE-Cy7-conjugated ant-NK1.1 (BioLegend), PE-Cy7-conjugated anti-CD8a (BioLegend), PE-CF594-conjugated anti-CD4 (BD Biosciences), and Alexa Fluor 488-conjugated anti-GPER (Santa Cruz Biotechnology, Santa Cruz, CA) with subsequent staining by Alexa Fluor 488-conjugated goat anti-rabbit immunoglobulin G (IgG) (Life Technologies). Then, the cells were washed, permeabilized with BD Cytofix/Cytoperm (BD Biosciences), and stained with PE-conjugated anti-IL-10 (BioLegend) to detect intracellular IL-10. *3)*, PE-Cy5-conjugated anti-TCRβ (BioLegend, San Diego, CA), PE-Cy7-conjugated ant-NK1.1 (BioLegend), PE-Cy7-conjugated anti-CD8a (BioLegend), and PE-CF594-conjugated anti-CD4 (BD Biosciences). Then, the cells were washed, permeabilized with BD Cytofix/Cytoperm (BD Biosciences), and stained with Alexa Fluor 488-conjugated IL-10 (BioLegend) to detect intracellular IL-10, and stained with PE-conjugated anti-Foxp3 (BioLegend) to direct intracellular Foxp3.

Multicolor flow analyses were performed using the Cytomics FC 500 (Beckman Coulter, Inc., Brea, CA) flow cytometer to allow for 5-color analysis. Acquired data were analyzed with FlowJo software (Tree Star, Inc., Ashland, OR).

### Statistical analysis

Two groups of data were compared using the Mann-Whitney U test. The P value for significance was set at less than 0.05. All results were expressed as the mean ± SEM.

## Results

### GPER expression in lung tissue

The expression of GPER in human immune cells (monocytes, macrophages, eosinophils, B cells, T cells) has been demonstrated [22,32]. GPER mRNA expression has been reported in the lungs of the C57BL/6 mouse strain [15,33]. We first examined the expression of GPER in the asthmatic lung tissue using immunofluorescence imaging. As shown in S1 Fig, GPER was expressed in the lung both in BALB/c and C57BL/6 mice.

### Administration of G-1 ameliorated AHR in BALB/c mice

**Fig 2:**
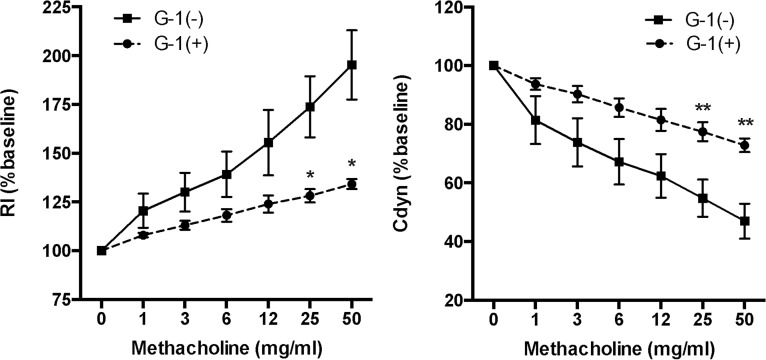
Decreased AHR in G-1-treated asthmatic BALB/c mice. Development of AHR was measured by increased lung resistance (Rl, left panel) and decreased dynamic lung compliance (Cdyn, right panel) in response to methacholine. AHR was significantly decreased in G-1-treated mice compared to controls. * P<0.05, ** P<0.01, G-1-treated (n = 6) vs. non-treated mice (n = 6).

Increased AHR induced by an immunologically non-specific stimulant such as methacholine is a fundamental feature of asthma. OVA-induced asthmatic mice develop AHR in response to increasing doses of inhaled methacholine. We examined the changes in airway resistance (reflecting AHR in large airways) and dynamic lung compliance (reflecting AHR in small airway) to assess whether G-1 administration influences AHR. As shown in Fig 2, in response to inhaled methacholine, asthmatic mice treated with phosphate buffered saline (PBS) had an increase in airway resistance and a decrease in lung compliance. In contrast, these responses to methacholine were significantly attenuated in G-1-treated asthmatic mice, indicating the suppression of AHR.

### Administration of G-1 attenuated inflammatory cell accumulation in BAL fluid in BALB/c mice

**Fig 3:**
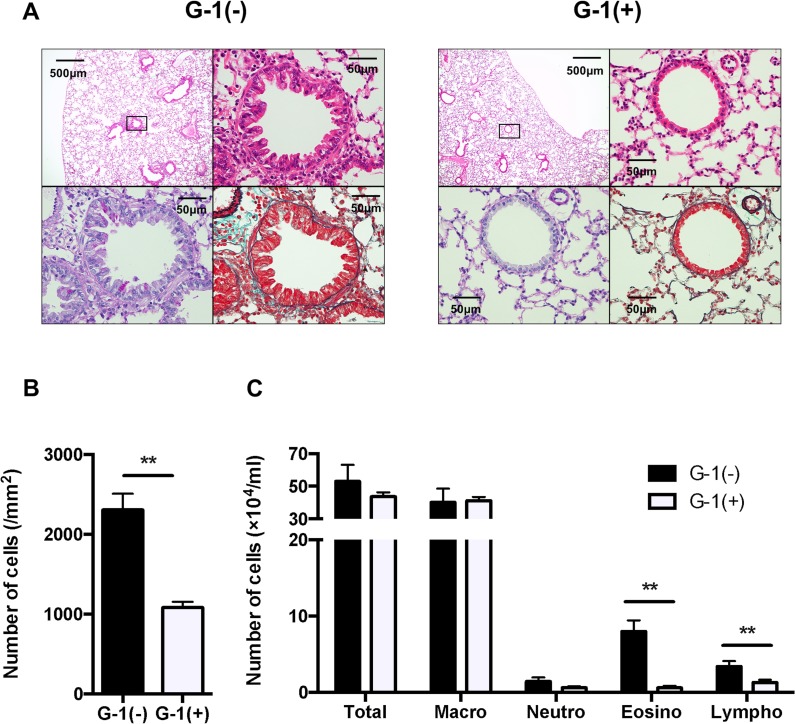
Inflammatory cell accumulation was significantly decreased in G-1-treated mice. *A*: H&E staining (original magnification: x40 and x400, upper panels) and PAS staining (x400, lower left panel) and MT staining (x400, lower right panel) of serial lung sections. The boxed area of the upper left panel is seen at higher magnification. *B*: Quantified data of inflammatory cell accumulation, histologically examined in H&E–stained lung tissue, as described in Materials and Methods. *C*: Inflammatory cell accumulation in BAL fluid. Values are expressed as the mean ± SEM. ** P<0.01, G-1-treated (n = 6) vs. non-treated mice (n = 6).

Since airway inflammation leads to development of AHR, we next investigated inflammatory cell infiltration into the airway by histological examination of H&E staining. In G-1-treated mice, inflammatory cells in the peribronchial areas were significantly decreased compared with those of non-treated BALB/c mice (Fig 3A, upper panels, Fig 3B). Goblet cells in airway epithelium and collagen deposition were also examined by PAS and MT staining, respectively. As shown in Fig 3A (lower panels), goblet cell hyperplasia and lung fibrosis were also attenuated in G-1-treated mice. The numbers and types of inflammatory cells in the airways were determined in BAL fluid 48 h after the last of the three consecutive allergen challenges (Fig 3C). Non-treated mice showed a marked increase in the number of eosinophils and lymphocytes in BAL fluid, whereas G-1 treatment significantly reduced the number of eosinophils and lymphocytes. These findings indicate that administration of G-1 suppress allergic airway inflammation.

### Administration of G-1 reduced levels of cytokine, chemokine, and OVA-specific IgE in BALB/c mice

**Fig 4:**
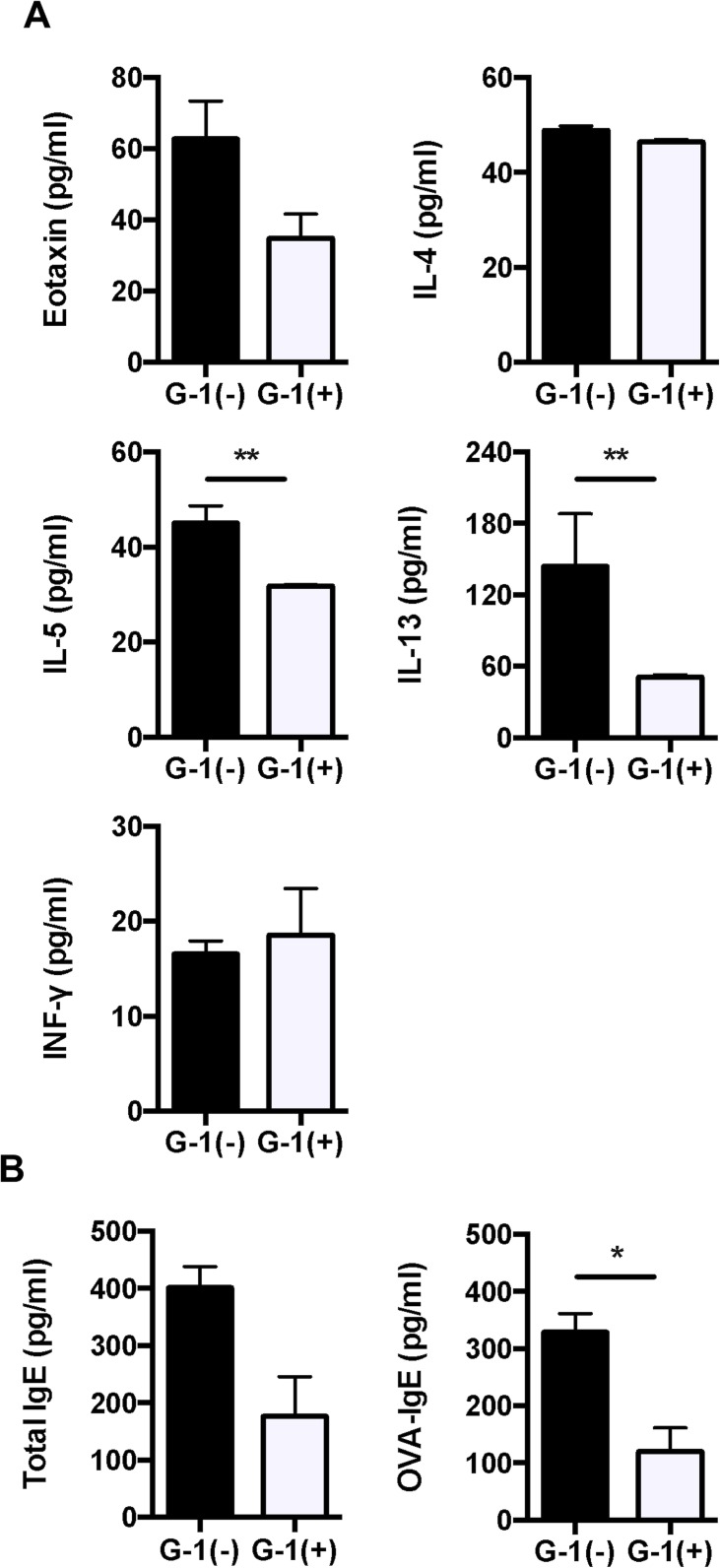
Th2 cytokines and IgE were decreased in G-1-treated BALB/c mice compared to non-treated controls. *A*, *B*: The levels of eotaxin, IL-4, IL-5, IL-13, and IFN-γ in BAL fluid and total and OVA-specific IgE antibodies in serum of mice were measured using ELISA. The results are expressed as the mean ± SEM. * P<0.05, ** P<0.01, G-1-treated (n = 6) vs. non-treated mice (n = 6).

Since G-1 attenuated OVA-induced lung inflammation, we measured levels of Th1 and Th2 cytokines (IL-4, 5, and 13 and INF-γ) and an eosinophil-driving chemokine eotaxin (CCL11) in BAL fluid using ELISA. Administration of G-1 significantly reduced the levels of IL-5 and IL-13 in BAL fluid compared with non-treated mice (Fig 4A). We also assessed the serum levels of total and OVA-specific IgE. As shown in Fig 4B, OVA-specific IgE was significantly reduced in G-1-treated asthmatic mice. These data suggest that G-1 administration attenuates Th2 cytokines in lung and antigen-specific B cell response in this asthmatic model of mice.

### Administration of G-1 attenuated allergic airway inflammation in C57BL/6 mice

**Fig 5:**
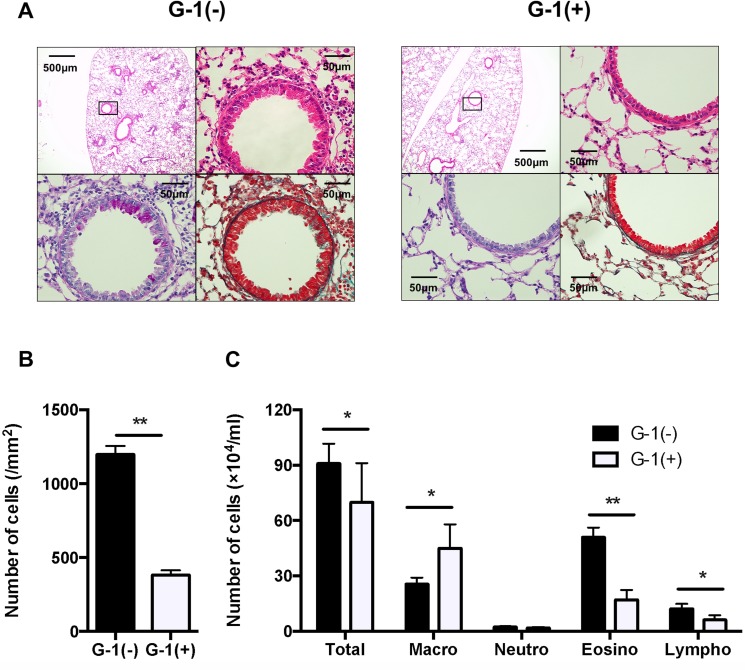
Inflammatory cell accumulation was significantly decreased in G-1-treated C57BL/6 mice compared to non-treated controls. *A*: H&E staining (original magnification: x40 and x400, upper panels) and PAS staining (x400, lower left panel) and MT staining (x400, lower right panel) of serial lung sections. *B*: Quantified data of inflammatory cell accumulation, histologically examined in H&E–stained lung tissue, as described in Materials and Methods. *C*: Inflammatory cell accumulation in BAL fluid. Values are expressed as the mean ± SEM. * P<0.05, ** P<0.01, G-1-treated (n = 6) vs. non-treated mice (n = 7).

**Fig 6:**
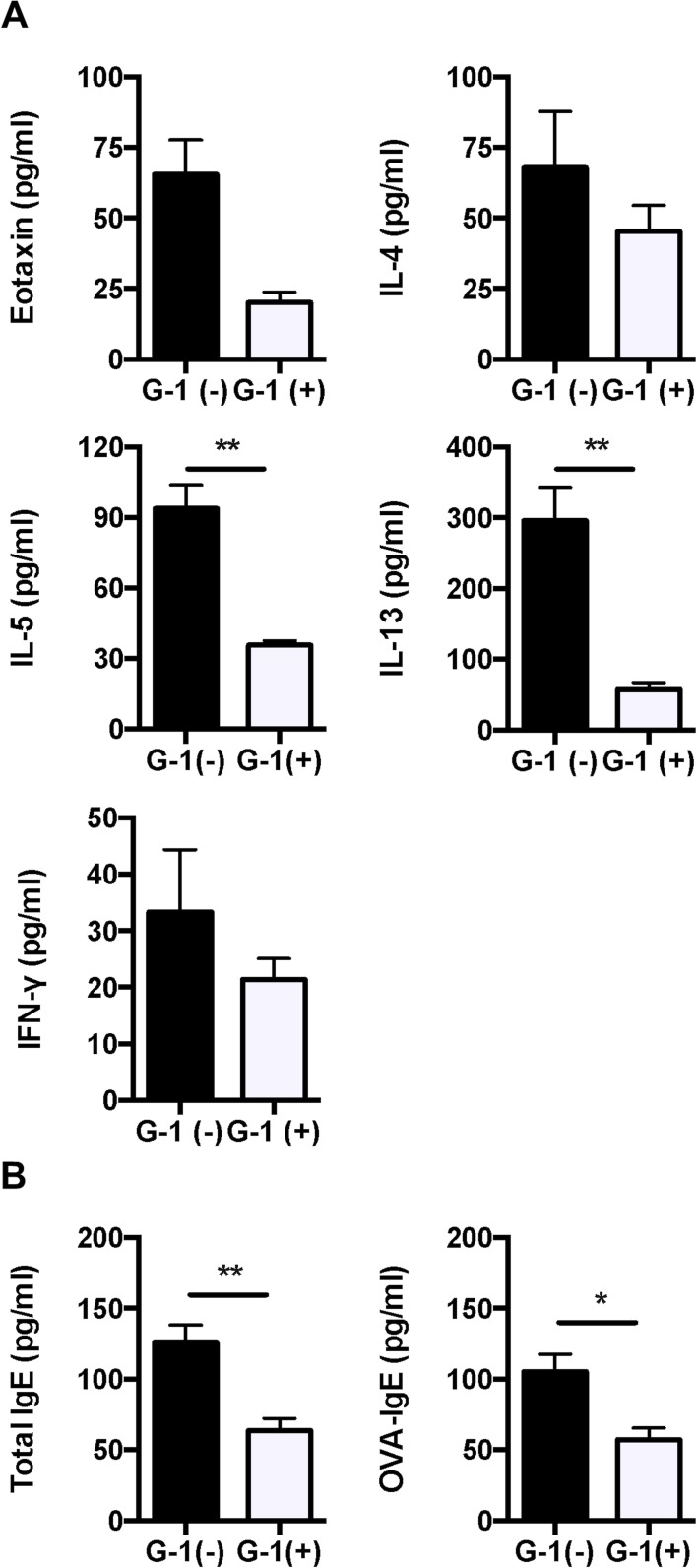
Th2 cytokines and IgE were decreased in G-1-treated C57BL/6 mice compared to non-treated mice. *A*, *B*: The levels of eotaxin, IL-4, IL-5, IL-13, and IFN-γ in BAL fluid and total and OVA-specific IgE antibodies in serum of mice were measured using ELISA. Values are expressed as the mean ± SEM. * P<0.05, ** P<0.01, G-1-treated (n = 6) vs. non-treated mice (n = 7).

BALB/c mice are thought to be immunologically Th2 shifted and thus used in asthmatic models more frequently than C57BL/6 mice. To examine whether the G-1-induced anti-inflammatory effects are limited to the Th2-shifted strain, we performed the same experiment using C57BL/6 mice. In H&E-stained lung sections, inflammatory cells in the peribronchial areas were significantly decreased in G-1-treated C57BL/6 mice (Fig 5A and 5B). PAS-stained goblet cells in airway epithelium and MT-stained collagen were decreased in G-1-treated mice (Fig 5A), similar to those in G-1-treated BALB/c mice (Fig 3A). G-1 administration significantly reduced the number of total cells, eosinophils, and lymphocytes in BAL fluids of C57BL/6 mice (Fig 5C). Administration of G-1 significantly reduced the levels of IL-5 and IL-13 in BAL fluid and serum levels of total and OVA-specific IgE antibodies in C57BL/6 mice, as well (Fig 6A and 6B). These findings indicate that the inhibition of allergic airway inflammation by G-1 is not dependent on the strain of mouse.

### IL-10 production was enhanced in splenocytes of G-1-treated C57BL/6 mice

**Fig 7:**
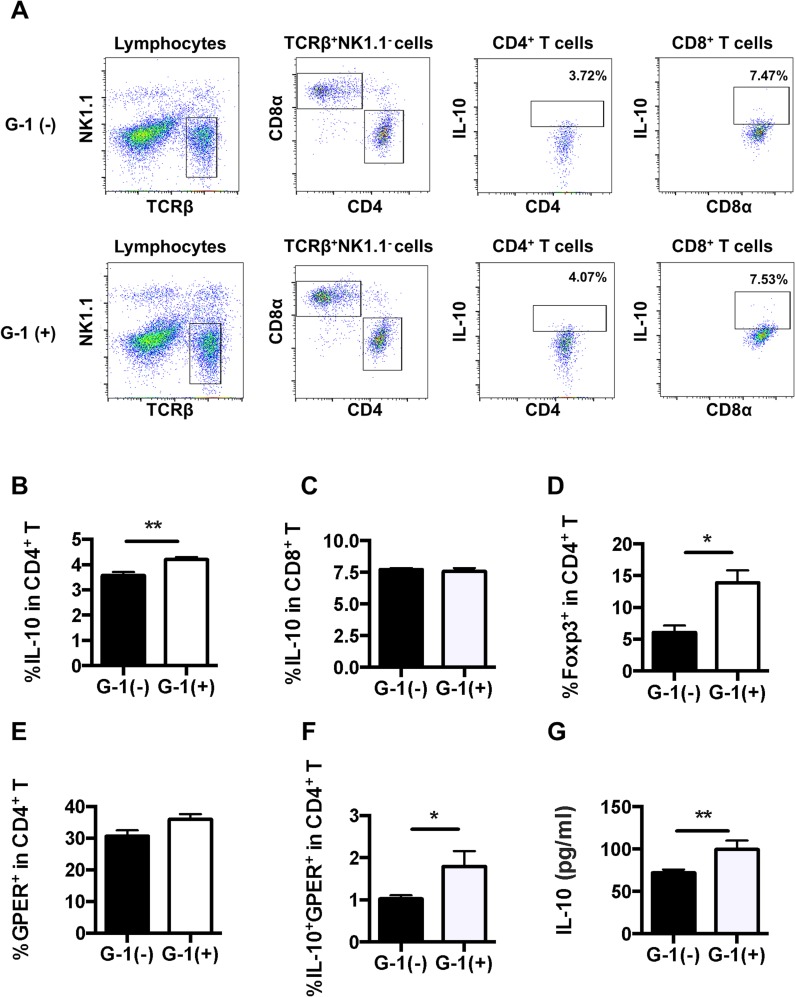
G-1 treatment increased the frequency of IL-10^+^CD4^+^ T cells and the secretion of IL-10 from splenocytes in C57BL/6 mice. *A*: Representative FACS data showing frequencies of IL-10^+^CD4^+^ T cells and IL-10^+^CD8^+^ T cells in splenocytes. *B*, *C*: Splenocytes were analyzed using a flow cytometer for the frequency of IL-10-producing CD4^+^ and CD8^+^ T cells. *D*: Splenocytes were analyzed using a flow cytometer for the frequency of Foxp3^+^CD4^+^ T cells. *E*, *F*: Splenocytes were analyzed using a flow cytometer for the frequency of GPER-expressing cells and IL-10-producing GPER^+^CD4^+^ T cells. *G*: The levels of IL-10 in splenocyte culture supernatant were measured by means of ELISA. Values are expressed as the mean ± SEM. * P<0.05, ** P<0.01, G-1-treated (n = 6) vs. non-treated mice (n = 7).

Since GPER has been reported to be associated with increased production of self-regulatory cytokine IL-10 [17,18], we hypothesized that IL-10 plays a pivotal role in G-1-induced anti-inflammatory effects. We first examined IL-10-producing CD4^+^ T cells and CD8^+^ T cells from freshly isolated spleens (Fig 7A). The frequency of IL-10^+^ CD4^+^ T cells was significantly higher in G-1-treated mice, although that of IL-10^+^ CD8^+^ T cells was comparable (Fig 7B and 7C). Foxp3^+^CD4^+^ regulatory T (Treg) cells have been shown to negatively regulate allergic airway inflammation through IL-10 [34]. Indeed, the frequency of Foxp3^+^CD4^+^ Treg cells was significantly higher in G-1-treated mice (Fig 7D). Further, splenic GPER^+^ CD4^+^ T cells and their IL-10 production were assessed. The frequency of GPER^+^CD4^+^ T cells was comparable (Fig 7E), although G-1-treated mice exhibited a significant increment of IL-10-producing populations in GPER^+^CD4^+^ T cells (Fig 7F). To confirm the upregulated IL-10 production from these cells, the culture supernatants were analyzed using ELISA (Fig 7D). Indeed, splenic mononuclear cells from G-1-treated mice produced a significantly higher amount of IL-10 as compared with the controls. These data indicate that G-1 enhances the IL-10 production from CD4^+^ T cell populations.

### Anti-inflammatory effects of G-1 were abolished in IL-10-deficient mice

**Fig 8:**
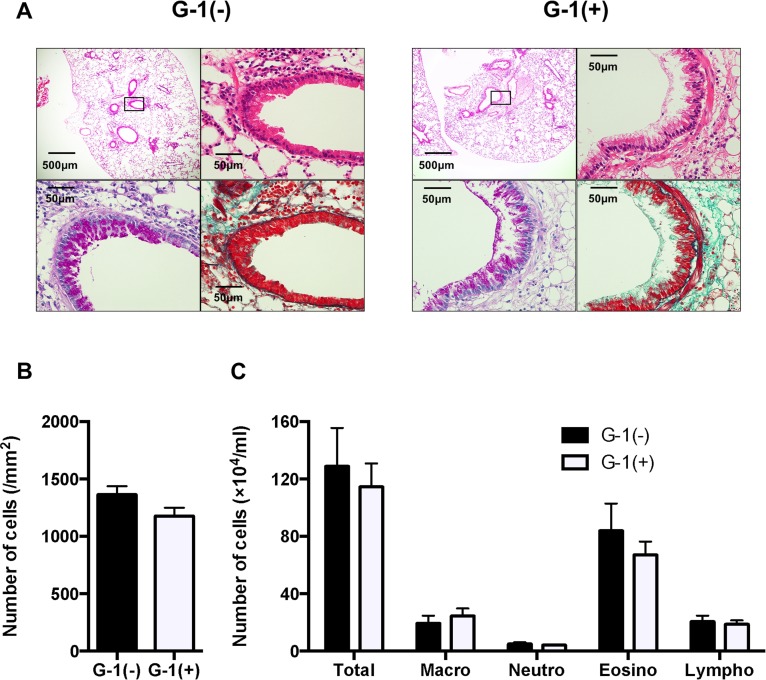
IL-10 deprivation abolished G-1-induced improvement of inflammatory cell accumulation in the lung. *A*: H&E staining (original magnification: x40 and x400, upper panels) and PAS staining (x400, lower left panel) and MT staining (x400, lower right panel) of serial lung sections. *B*: Quantified data of inflammatory cell accumulation, histologically examined in H&E–stained lung tissue, as described in Materials and Methods. *C*: Inflammatory cell accumulation in BAL fluid. Values are expressed as the mean ± SEM for G-1-treated mice (n = 6) and non-treated controls (n = 7).

Since IL-10 production was upregulated in splenocytes from G-1-treated mice, we examined G-1-treated lung inflammation in IL-10 KO mice with a C57BL/6 background. Histopathological examination demonstrated no significant differences in inflammatory cell accumulation, goblet cell hyperplasia, and lung fibrosis between G-1-treated and non-treated IL-10 KO mice (Fig 8A and 8B). In addition, the numbers and types of inflammatory cells in the airways did not differ regardless of G-1 treatment (Fig 8C).

**Fig 9:**
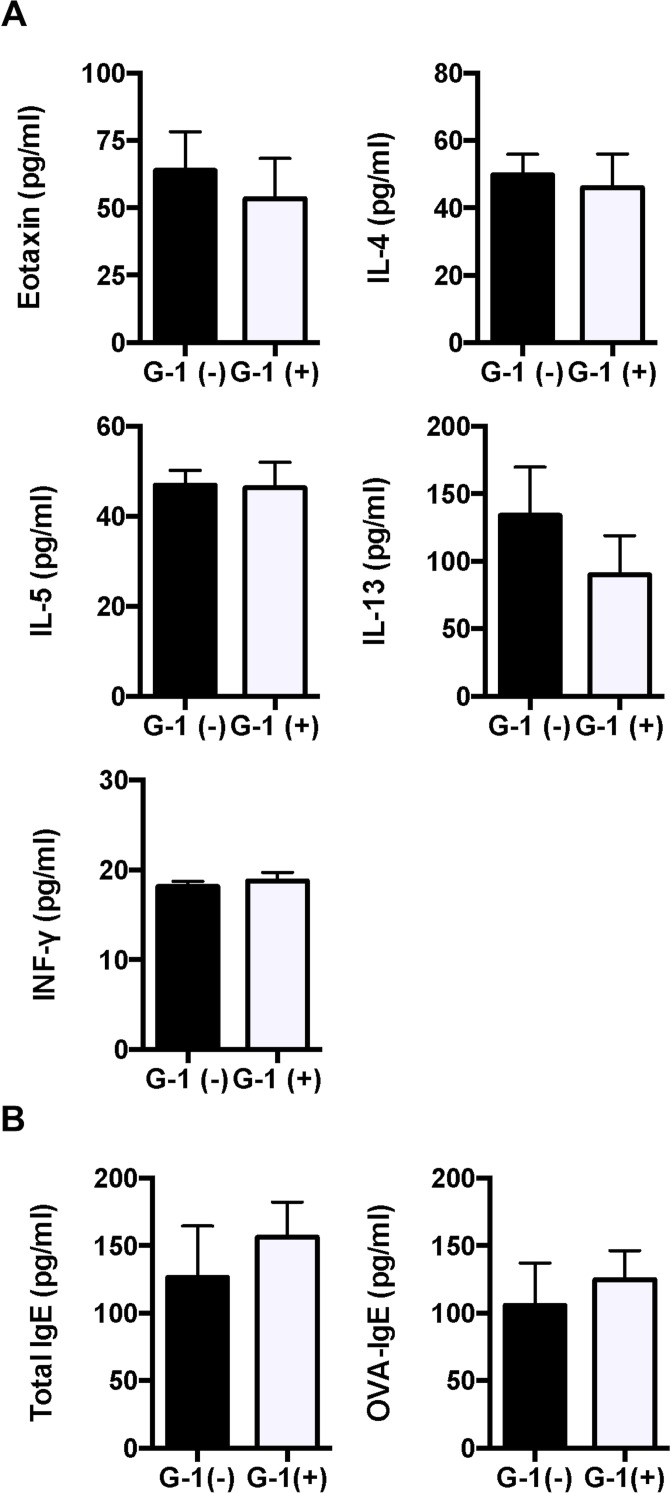
IL-10 depletion eradicated G-1-induced reduction of Th2 cytokines in BAL fluid and serum IgE. *A*, *B*: The levels of eotaxin, IL-4, IL-5, IL-10, IL-13, and IFN-γ in BAL fluid and total and OVA-specific IgE antibodies in serum of IL-10 KO mice were measured using ELISA. Values are expressed as the mean ± SEM for G-1-treated mice (n = 6) and non-treated controls (n = 7).

Concentrations of cytokines (IL-4, 5, and 13 and INF-γ) and a chemokine (eotaxin) in BAL fluid were measured using ELISA. Although IL-5, IL-13, and OVA-specific IgE antibodies were reduced in G-1-treated wild-type mice (Fig 6A and 6B), there were no differences in these between G-1-treated and non-treated IL-10 KO mice (Fig 9A). Moreover, G-1 treatment did not reduce serum levels of total or OVA-specific IgE in IL-10-deprived mice (Fig 9B). These findings indicate that the G-1-induced suppressive effect on allergic airway inflammation is dependent on the IL-10 signaling pathway.

## Discussion

Understanding the mechanisms by which estrogen influences asthma is of paramount importance, especially for better treatment of female asthmatics. Here, we demonstrated that administration of a GPER-specific agonist during the allergen challenge phase inhibited allergic airway response in two different strains of mice, i.e., those with BALB/c and C57BL/6 background. The effect was associated with increased splenic CD4^+^ T cells that produce IL-10. IL-10 plays an indispensable role in the effect of G-1, evidenced by the fact that the changes in airway inflammation and cytokine production were not observed in IL-10 KO asthmatic mice. To the best of our knowledge, this is the first demonstration of the involvement of GPER in allergic airway inflammation.

Estrogen in the allergic inflammatory process is a complex phenomenon with both pro- and anti-inflammatory effects. Estradiol can activate the inflammatory cells including mast cells and eosinophils [35,36], although it reduces airway constriction [37,38], down-regulates the production of pro-inflammatory cytokines [39], and protects the cells from harmful oxidative stress [40]. The influence of estrogen on asthma has been investigated in rodent models with exogenous estradiol administration and ovariectomy. The results have shown that estrogen plays unfavorable [7,41], favorable [42], and dual [43] roles in allergic airway inflammatory response. The inconsistency is likely due to different receptors and signaling pathways, which can be altered depending on cell type, and other confounding factors. Recent discovery of GPER and studies using the specific synthetic agonist G-1 and GPER KO mice revealed differential effects of ER and GPER [16]. The current study protocol was aimed at examining the role of GPER activation during the allergen challenge phase using an established asthmatic model.

The improvement of AHR by G-1 is associated with reduced airway inflammation. The direct effect of G-1 on airway smooth muscle cells to induce airway relaxation is not likely, because a recent report indicated no effect of G-1 on tracheal relaxation using *ex vivo* experiments [37]. The first step of leukocyte migration from the bloodstream into inflamed tissue starts with adhesion to the endothelium by interaction of integrins on leukocytes with upregulated adhesion molecules of the immunoglobulin family, such as intercellular adhesion molecule-1 (ICAM-1) and vascular cell adhesion molecule-1 (VCAM-1) [44,45]. Leukocytes are subsequently recruited in a stepwise manner involving rolling, activation, firm adhesion, and transmigration from the blood stream into extravascular tissues, further contributing to inflammation. Chakrabarti *et al*. reported that G-1 down-regulated the expression of these adhesion molecules in endothelial cells [45]. GPER-dependent down-regulation of the adhesion molecules provides a possible explanation for suppressed lung inflammation in G-1-treated mice.

The finding that G-1 treatment reduced the production of IL-5 and IL-13 in two different mice strains is of particular importance because these cytokines have been closely linked to allergic inflammation. IL-5 is the principal cytokine that modulates eosinophil function. During an allergic response, IL-5 stimulates the differentiation of eosinophils from bone marrow cells and maintains cell survival resulting in blood eosinophilia [46]. At the site of inflammation, IL-5 prolongs eosinophil survival, and eosinophils release cytotoxic products including granular proteins and oxygen radicals leading to AHR [47]. IL-13, which shares a receptor component and signaling pathways with IL-4, plays a central role in airway eosinophilia and development of AHR [48]. Since IL-13 directs IgE class-switching in naive B cells [49], IL-13 likely contributes to decreasing IgE production in G-1-treated mice. In contrast to other Th2 lymphocyte-derived cytokines, IL-4 was not affected by G-1 treatment in this study. Recently, studies have identified a family of lineage-negative innate lymphoid cells (ILCs). Among them, type 2 ILCs (ILC2) are known to produce abundant amounts of IL-5 and IL-13 in the lung [50,51]. The relationship between ILC2 and GPER is a subject for future study.

IL-10, primarily a Th2 product, is an intrinsic self-regulatory cytokine in allergic conditions [52]. IL-10 has been known to dampen antigen-specific T cell responses such as cytokine production and proliferation [53,54]. Estradiol has been reported to protect against development of experimental autoimmune encephalomyelitis (EAE) in mice by decreasing the production of inflammatory cytokines and increasing IL-10, and through expansion of Treg cells [55,56]. A recent study revealed that GPER played a critical role in increased production of IL-10 in this system [17]. Consistent with two independent reports [18,20], we showed an increase in IL-10 secretion from splenocytes isolated from G-1-treated mice. G-1 has been shown to elicit IL-10 expression in Th17-polarized CD4^+^ T cells, increasing the number of IL-10 and IL-17 double positive cells via *de novo* IL-10 induction [18]. Our current data indicate that systemic administration of G-1 increases the Treg cells. Indeed, Wang *et al*. reported that G-1 enhanced suppressive activity of CD4^+^Foxp3^+^ Treg cells through programmed cell death-1 (PD-1) in a GPER-dependent manner [20]. Although we could not use a littermate control, the lack of inhibitory effects of G-1 in IL-10 KO mice was clearly observed. Taken together, GPER activation controls the acute asthmatic condition by increasing the lymphocyte populations that produce IL-10, resulting in diminished airway inflammation.

In summary, in the present experimental model of acute asthma, allergic response was regulated by administration of a GPER-specific agonist. The relative increase in IL-10 production by extended GPER activation limits the production of allergenic cytokines such as IL-5 and IL-13, as well as the IgE production and accumulation of inflammatory cells. The current study provides novel insights into the functional mechanism of estrogen in asthmatics. Given the implication of GPER, especially in female reproductive cancer, GPER is now considered to be an important target of drug development. In this context, the current study also highlights a potential role of GPER as a novel therapeutic target for future treatments of asthma.

## Supporting Information

S1 Fig Immunofluorescence staining demonstrated GPER expression in asthmatic lung tissue in BALB/c (A) and C57BL/6 (B) mice. The sections were blocked in blocking buffer (3% bovine serum albumin (BSA) in PBS) for 1 hour and incubated with anti-G-protein-coupled estrogen receptor antibodies (GPR30 (N-15)-R: sc-48525-R; rabbit polyclonal, 1:50; SANTA CRUZ, Dallas, TX), diluted in PBS at room temperature for 2 hours. Subsequently, the sections were rinsed in PBS, incubated with Alexa Fluor 488 goat anti-rabbit IgG (1:200, Invitrogen, Grand Island, NY), and counterstained with Hoechst 33342 and trihydrochloride trihydrate (1:5000, Invitrogen). The slides were analyzed using a confocal microscope (Carl Zeiss LSM510). fluorescein isothiocyanate (FITC) (green) was used to visualize GPER, whereas Hoechst 33342 (blue) was used for nuclear staining.
(TIFF)

## References

[1] The ENFUMOSA Study Group (2003) The ENFUMOSA cross-sectional European multicentre study of the clinical phenotype of chronic severe asthma. European Network for Understanding Mechanisms of Severe Asthma. Eur Respir J 22: 470–477. 14516137 10.1183/09031936.03.00261903

[2] Skobeloff EM, Spivey WH, St Clair SS, Schoffstall JM (1992) The influence of age and sex on asthma admissions. JAMA 268: 3437–3440. 1460733

[3] Leynaert B, Bousquet J, Henry C, Liard R, Neukirch F (1997) Is bronchial hyperresponsiveness more frequent in women than in men? A population-based study. Am J Respir Crit Care Med 156: 1413–1420. 9372654 10.1164/ajrccm.156.5.9701060

[4] Okuyama K, Wada K, Chihara J, Takayanagi M, Ohno I (2008) Sex-related splenocyte function in a murine model of allergic asthma. Clin Exp Allergy 38: 1212–1219. 10.1111/j.1365-2222.2008.03015.x 18498415

[5] Takeda M, Tanabe M, Ito W, Ueki S, Konnno Y, Chihara M, et al (2013) Gender difference in allergic airway remodelling and immunoglobulin production in mouse model of asthma. Respirology 18: 797–806. 10.1111/resp.12078 23490273

[6] Moore WC, Meyers DA, Wenzel SE, Teague WG, Li H, Li X, et al (2010) Identification of asthma phenotypes using cluster analysis in the Severe Asthma Research Program. Am J Respir Crit Care Med 181: 315–323. 10.1164/rccm.200906-0896OC 19892860 PMC2822971

[7] Cai Y, Zhou J, Webb DC (2012) Estrogen stimulates Th2 cytokine production and regulates the compartmentalisation of eosinophils during allergen challenge in a mouse model of asthma. Int Arch Allergy Immunol 158: 252–260. 10.1159/000331437 22398379

[8] Carey MA, Card JW, Voltz JW, Arbes SJ Jr., Germolec DR, Korach KS, et al (2007) It's all about sex: gender, lung development and lung disease. Trends Endocrinol Metab 18: 308–313. 17764971 10.1016/j.tem.2007.08.003 PMC2391086

[9] Myers JR, Sherman CB (1994) Should supplemental estrogens be used as steroid-sparing agents in asthmatic women? Chest 106: 318–319. 8020306 10.1378/chest.106.1.318

[10] Ensom MH, Chong G, Beaudin B, Bai TR (2003) Estradiol in severe asthma with premenstrual worsening. Ann Pharmacother 37: 1610–1613. 14565797 10.1345/aph.1D090

[11] Lim RH, Kobzik L (2008) Sexual tension in the airways: the puzzling duality of estrogen in asthma. Am J Respir Cell Mol Biol 38: 499–500. 10.1165/rcmb.2008-0002ED 18417757

[12] Pietras RJ, Szego CM (1975) Endometrial cell calcium and oestrogen action. Nature 253: 357–359. 1167402 10.1038/253357a0

[13] Pietras RJ, Szego CM (1977) Specific binding sites for oestrogen at the outer surfaces of isolated endometrial cells. Nature 265: 69–72. 834244 10.1038/265069a0

[14] Feng Y, Gregor P (1997) Cloning of a novel member of the G protein-coupled receptor family related to peptide receptors. Biochem Biophys Res Commun 231: 651–654. 9070864 10.1006/bbrc.1997.6161

[15] Isensee J, Meoli L, Zazzu V, Nabzdyk C, Witt H, Soewarto D, et al (2009) Expression pattern of G protein-coupled receptor 30 in LacZ reporter mice. Endocrinology 150: 1722–1730. 10.1210/en.2008-1488 19095739

[16] Filardo EJ, Thomas P (2012) Minireview: G protein-coupled estrogen receptor-1, GPER-1: its mechanism of action and role in female reproductive cancer, renal and vascular physiology. Endocrinology 153: 2953–2962. 10.1210/en.2012-1061 22495674 PMC3380306

[17] Yates MA, Li Y, Chlebeck PJ, Offner H (2010) GPR30, but not estrogen receptor-alpha, is crucial in the treatment of experimental autoimmune encephalomyelitis by oral ethinyl estradiol. BMC Immunol 11: 20 10.1186/1471-2172-11-20 20403194 PMC2864220

[18] Brunsing RL, Prossnitz ER (2011) Induction of interleukin-10 in the T helper type 17 effector population by the G protein coupled estrogen receptor (GPER) agonist G-1. Immunology 134: 93–106. 10.1111/j.1365-2567.2011.03471.x 21722102 PMC3173698

[19] Blasko E, Haskell CA, Leung S, Gualtieri G, Halks-Miller M, Mahmoudi M, et al (2009) Beneficial role of the GPR30 agonist G-1 in an animal model of multiple sclerosis. J Neuroimmunol 214: 67–77. 10.1016/j.jneuroim.2009.06.023 19664827 PMC2873862

[20] Wang C, Dehghani B, Li Y, Kaler LJ, Proctor T, Vandenbark AA, et al (2009) Membrane estrogen receptor regulates experimental autoimmune encephalomyelitis through up-regulation of programmed death 1. J Immunol 182: 3294–3303. 10.4049/jimmunol.0803205 19234228 PMC2729563

[21] Windahl SH, Andersson N, Chagin AS, Martensson UE, Carlsten H, Olde B, et al (2009) The role of the G protein-coupled receptor GPR30 in the effects of estrogen in ovariectomized mice. Am J Physiol Endocrinol Metab 296: E490–496. 10.1152/ajpendo.90691.2008 19088255

[22] Tamaki M, Konno Y, Kobayashi Y, Takeda M, Itoga M, Moritoki Y, et al (2014) Expression and functional roles of G-protein-coupled estrogen receptor (GPER) in human eosinophils. Immunol Lett 160: 72–78. 10.1016/j.imlet.2014.03.012 24718279

[23] Lahn M, Kanehiro A, Takeda K, Joetham A, Schwarze J, Kohler G, et al (1999) Negative regulation of airway responsiveness that is dependent on gammadelta T cells and independent of alphabeta T cells. Nat Med 5: 1150–1156. 10502818 10.1038/13476

[24] Kanehiro A, Takeda K, Joetham A, Tomkinson A, Ikemura T, Irvin CG, et al (2000) Timing of administration of anti-VLA-4 differentiates airway hyperresponsiveness in the central and peripheral airways in mice. Am J Respir Crit Care Med 162: 1132–1139. 10988142 10.1164/ajrccm.162.3.9910100

[25] Bologa CG, Revankar CM, Young SM, Edwards BS, Arterburn JB, Kiselyov AS, et al (2006) Virtual and biomolecular screening converge on a selective agonist for GPR30. Nat Chem Biol 2: 207–212. 16520733 10.1038/nchembio775

[26] Engdahl C, Jochems C, Windahl SH, Borjesson AE, Ohlsson C, Carlsten H, et al (2010) Amelioration of collagen-induced arthritis and immune-associated bone loss through signaling via estrogen receptor alpha, and not estrogen receptor beta or G protein-coupled receptor 30. Arthritis Rheum 62: 524–533. 10.1002/art.25055 20112355

[27] Hammond R, Nelson D, Gibbs RB (2011) GPR30 co-localizes with cholinergic neurons in the basal forebrain and enhances potassium-stimulated acetylcholine release in the hippocampus. Psychoneuroendocrinology 36: 182–192. 10.1016/j.psyneuen.2010.07.007 20696528 PMC2994977

[28] Lai WQ, Goh HH, Bao Z, Wong WS, Melendez AJ, Leung BP (2008) The role of sphingosine kinase in a murine model of allergic asthma. J Immunol 180: 4323–4329. 18322246 10.4049/jimmunol.180.6.4323

[29] Lee KS, Lee HK, Hayflick JS, Lee YC, Puri KD (2006) Inhibition of phosphoinositide 3-kinase delta attenuates allergic airway inflammation and hyperresponsiveness in murine asthma model. FASEB J 20: 455–465. 16507763 10.1096/fj.05-5045com

[30] Kuhn C 3rd, Homer RJ, Zhu Z, Ward N, Flavell RA, Geba GP, et al (2000) Airway hyperresponsiveness and airway obstruction in transgenic mice. Morphologic correlates in mice overexpressing interleukin (IL)-11 and IL-6 in the lung. Am J Respir Cell Mol Biol 22: 289–295. 10696065 10.1165/ajrcmb.22.3.3690

[31] Wise JT, Baginski TJ, Mobley JL (1999) An adoptive transfer model of allergic lung inflammation in mice is mediated by CD4+CD62LlowCD25+ T cells. J Immunol 162: 5592–5600. 10228042

[32] Kvingedal AM, Smeland EB (1997) A novel putative G-protein-coupled receptor expressed in lung, heart and lymphoid tissue. FEBS Lett 407: 59–62. 9141481 10.1016/s0014-5793(97)00278-0

[33] Martensson UE, Salehi SA, Windahl S, Gomez MF, Sward K, Daszkiewicz-Nilsson J, et al (2009) Deletion of the G protein-coupled receptor 30 impairs glucose tolerance, reduces bone growth, increases blood pressure, and eliminates estradiol-stimulated insulin release in female mice. Endocrinology 150: 687–698. 10.1210/en.2008-0623 18845638

[34] Rubtsov YP, Rasmussen JP, Chi EY, Fontenot J, Castelli L, Ye X, et al (2008) Regulatory T cell-derived interleukin-10 limits inflammation at environmental interfaces. Immunity 28: 546–558. 10.1016/j.immuni.2008.02.017 18387831

[35] Jensen F, Woudwyk M, Teles A, Woidacki K, Taran F, Costa S, et al (2010) Estradiol and progesterone regulate the migration of mast cells from the periphery to the uterus and induce their maturation and degranulation. PLoS One 5: e14409 10.1371/journal.pone.0014409 21203555 PMC3008683

[36] Hamano N, Terada N, Maesako K, Numata T, Konno A (1998) Effect of sex hormones on eosinophilic inflammation in nasal mucosa. Allergy Asthma Proc 19: 263–269. 9801739 10.2500/108854198778557773

[37] Intapad S, Dimitropoulou C, Snead C, Piyachaturawat P, Catravas JD (2012) Regulation of asthmatic airway relaxation by estrogen and heat shock protein 90. J Cell Physiol 227: 3036–3043. 10.1002/jcp.23045 22016308

[38] Dimitropoulou C, White RE, Ownby DR, Catravas JD (2005) Estrogen reduces carbachol-induced constriction of asthmatic airways by stimulating large-conductance voltage and calcium-dependent potassium channels. Am J Respir Cell Mol Biol 32: 239–247. 15626773 10.1165/rcmb.2004-0331OC

[39] Kramer PR, Kramer SF, Guan G (2004) 17 beta-estradiol regulates cytokine release through modulation of CD16 expression in monocytes and monocyte-derived macrophages. Arthritis Rheum 50: 1967–1975. 15188374 10.1002/art.20309

[40] Strehlow K, Rotter S, Wassmann S, Adam O, Grohe C, Laufs K, et al (2003) Modulation of antioxidant enzyme expression and function by estrogen. Circ Res 93: 170–177. 12816884 10.1161/01.RES.0000082334.17947.11

[41] de Oliveira AP, Domingos HV, Cavriani G, Damazo AS, Dos Santos Franco AL, Oliani SM, et al (2007) Cellular recruitment and cytokine generation in a rat model of allergic lung inflammation are differentially modulated by progesterone and estradiol. Am J Physiol Cell Physiol 293: C1120–1128. 17634417 10.1152/ajpcell.00286.2006

[42] Dimitropoulou C, Drakopanagiotakis F, Chatterjee A, Snead C, Catravas JD (2009) Estrogen replacement therapy prevents airway dysfunction in a murine model of allergen-induced asthma. Lung 187: 116–127. 10.1007/s00408-008-9129-z 19083056

[43] Riffo-Vasquez Y, Ligeiro de Oliveira AP, Page CP, Spina D, Tavares-de-Lima W (2007) Role of sex hormones in allergic inflammation in mice. Clin Exp Allergy 37: 459–470. 17359396 10.1111/j.1365-2222.2007.02670.x

[44] Yamamoto R, Ueki S, Moritoki Y, Kobayashi Y, Oyamada H, Konno Y, et al (2013) Adiponectin attenuates human eosinophil adhesion and chemotaxis: implications in allergic inflammation. J Asthma 50: 828–835. 10.3109/02770903.2013.816725 23777560

[45] Chakrabarti S, Davidge ST (2012) G-protein coupled receptor 30 (GPR30): a novel regulator of endothelial inflammation. PLoS One 7: e52357 10.1371/journal.pone.0052357 23285008 PMC3527521

[46] Rothenberg ME, Hogan SP (2006) The eosinophil. Annu Rev Immunol 24: 147–174. 16551246 10.1146/annurev.immunol.24.021605.090720

[47] Ueki S, Adachi T, Bourdeaux J, Oyamada H, Yamada Y, Hamada K, et al (2003) Expression of PPARgamma in eosinophils and its functional role in survival and chemotaxis. Immunol Lett 86: 183–189. 12644321 10.1016/s0165-2478(03)00003-8

[48] Wills-Karp M, Luyimbazi J, Xu X, Schofield B, Neben TY, Karp CL, et al (1998) Interleukin-13: central mediator of allergic asthma. Science 282: 2258–2261. 9856949 10.1126/science.282.5397.2258

[49] Aversa G, Punnonen J, Cocks BG, de Waal Malefyt R, Vega F Jr., Zurawski SM, et al (1993) An interleukin 4 (IL-4) mutant protein inhibits both IL-4 or IL-13-induced human immunoglobulin G4 (IgG4) and IgE synthesis and B cell proliferation: support for a common component shared by IL-4 and IL-13 receptors. J Exp Med 178: 2213–2218. 7504061 10.1084/jem.178.6.2213 PMC2191286

[50] Li BW, Hendriks RW (2013) Group 2 innate lymphoid cells in lung inflammation. Immunology 140: 281–287. 10.1111/imm.12153 23866009 PMC3800433

[51] Klein Wolterink RG, Kleinjan A, van Nimwegen M, Bergen I, de Bruijn M, Levani Y, et al (2012) Pulmonary innate lymphoid cells are major producers of IL-5 and IL-13 in murine models of allergic asthma. Eur J Immunol 42: 1106–1116. 10.1002/eji.201142018 22539286

[52] Fiorentino DF, Bond MW, Mosmann TR (1989) Two types of mouse T helper cell. IV. Th2 clones secrete a factor that inhibits cytokine production by Th1 clones. J Exp Med 170: 2081–2095. 2531194 10.1084/jem.170.6.2081 PMC2189521

[53] Zuany-Amorim C, Creminon C, Nevers MC, Nahori MA, Vargaftig BB, Pretolani M (1996) Modulation by IL-10 of antigen-induced IL-5 generation, and CD4+ T lymphocyte and eosinophil infiltration into the mouse peritoneal cavity. J Immunol 157: 377–384. 8683140

[54] Del Prete G, De Carli M, Almerigogna F, Giudizi MG, Biagiotti R, Romagnani S (1993) Human IL-10 is produced by both type 1 helper (Th1) and type 2 helper (Th2) T cell clones and inhibits their antigen-specific proliferation and cytokine production. J Immunol 150: 353–360. 8419468

[55] Polanczyk MJ, Hopke C, Vandenbark AA, Offner H (2007) Treg suppressive activity involves estrogen-dependent expression of programmed death-1 (PD-1). Int Immunol 19: 337–343. 17267414 10.1093/intimm/dxl151

[56] Polanczyk MJ, Carson BD, Subramanian S, Afentoulis M, Vandenbark AA, Ziegler SF, et al (2004) Cutting edge: estrogen drives expansion of the CD4+CD25+ regulatory T cell compartment. J Immunol 173: 2227–2230. 15294932 10.4049/jimmunol.173.4.2227

