# Correction: G-Protein-Coupled Estrogen Receptor Agonist Suppresses Airway Inflammation in a Mouse Model of Asthma through IL-10

**DOI:** 10.1371/journal.pone.0136326

**Published:** 2015-08-14

**Authors:** Masamichi Itoga, Yasunori Konno, Yuki Moritoki, Yukiko Saito, Wataru Ito, Mami Tamaki, Yoshiki Kobayashi, Hiroyuki Kayaba, Yuta Kikuchi, Junichi Chihara, Masahide Takeda, Shigeharu Ueki, Makoto Hirokawa

In the Materials and Methods section, there is an error in the fifth and sixth sentences of the section titled “Flow cytometry.” This sentence should read: 2), PE-Cy5-conjugated anti-TCRβ (BioLegend, San Diego, CA), PE-Cy7-conjugated ant-NK1.1 (BioLegend), PE-Cy7-conjugated anti-CD8a (BioLegend), PE-CF594-conjugated anti-CD4 (BD Biosciences), and GPR30 (N-15)-R Antibody (Santa Cruz Biotechnology, Santa Cruz, CA) with subsequent staining by Alexa Fluor 488-conjugated goat anti-rabbit immunoglobulin G (IgG) (Invitrogen, Grand Island, NY). Then, the cells were washed, permeabilized with BD Cytofix/Cytoperm (BD Biosciences), and stained with PE-conjugated anti-IL-10 (BioLegend) to detect intracellular IL-10.
