# Supplementary figures and images for: G-Protein-Coupled Estrogen Receptor Agonist Suppresses Airway Inflammation in a Mouse Model of Asthma through IL-10
Source: PLoS One. 2015 Mar 31;10(3):e0123210. doi: 10.1371/journal.pone.0123210 (PMC4380451; doi:10.1371/journal.pone.0123210)

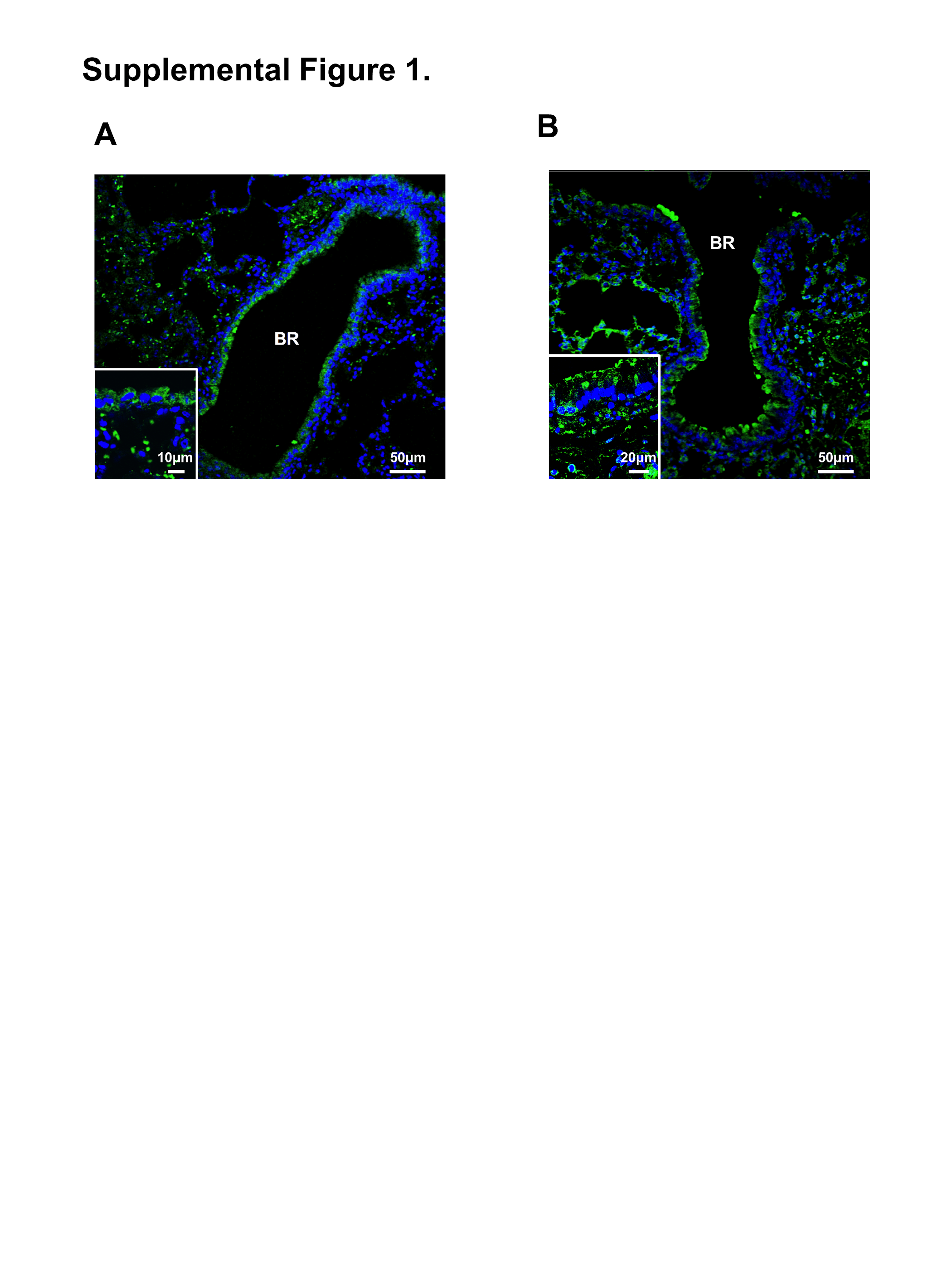

Supplement: S1 Fig — The sections were blocked in blocking buffer (3% bovine serum albumin (BSA) in PBS) for 1 hour and incubated with anti-G-protein-coupled estrogen receptor antibodies (GPR30 (N-15)-R: sc-48525-R; rabbit polyclonal, 1:50; SANTA CRUZ, Dallas, TX), diluted in PBS at room temperature for 2 hours. Subsequently, the sections were rinsed in PBS, incubated with Alexa Fluor 488 goat anti-rabbit IgG (1:200, Invitrogen, Grand Island, NY), and counterstained with Hoechst 33342 and trihydrochloride trihydrate (1:5000, Invitrogen). The slides were analyzed using a confocal microscope (Carl Zeiss LSM510). fluorescein isothiocyanate (FITC) (green) was used to visualize GPER, whereas Hoechst 33342 (blue) was used for nuclear staining. (TIFF) [file pone.0123210.s001.tiff]
